# Impact of telehealth on hospital use and mortality: provisional findings from the whole system demonstrator trial

**Published:** 2012-06-15

**Authors:** Adam Steventon, John Billings, Jennifer Dixon, Martin Bardsley

**Affiliations:** The Nuffield Trust, UK; NYU Wagner, USA; The Nuffield Trust, UK; The Nuffield Trust, UK

**Keywords:** telehealth, evaluation, primary care, social care

## Abstract

**Introduction:**

Around the world, efforts are being made to address the increasing prevalence of chronic disease among an ageing population. Some research has suggested that telehealth can improve patient experience, clinical indicators and quality of life, while lowering use of traditional health services. However results are equivocal and many trials have not met robust evaluation standards.

**Aims and objectives:**

The whole system demonstrator was a large cluster randomised trial aimed at a broad assessment of the impact of telehealth on a population with Chronic Obstructive Pulmonary Disease (COPD), heart failure or diabetes. This study considers the impact of telehealth on services other than hospital care, including primary care and social care.

**Methods:**

Novel data linkage techniques aimed to allow participants to be tracked in data sets collected from several hundred general practices and three local authorities. The outputs from a predictive model were used to adjust for differences in case mix between intervention and control groups. We assessed the impact of telehealth on numbers of visits to general practice surgeries, numbers of hours of home care from the local authority, and admission to residential care over twelve months.

**Results:**

Under embargo.

**Conclusions:**

Under embargo.

